# Supplementary figures and images for: Long-Lasting, Fine-Tuned Anti-Tumor Activity of Recombinant Listeria monocytogenes Vaccine Is Controlled by Pyroptosis and Necroptosis Regulatory and Effector Molecules
Source: Pathogens. 2024 Sep 25;13(10):828. doi: 10.3390/pathogens13100828 (PMC11510422; doi:10.3390/pathogens13100828)

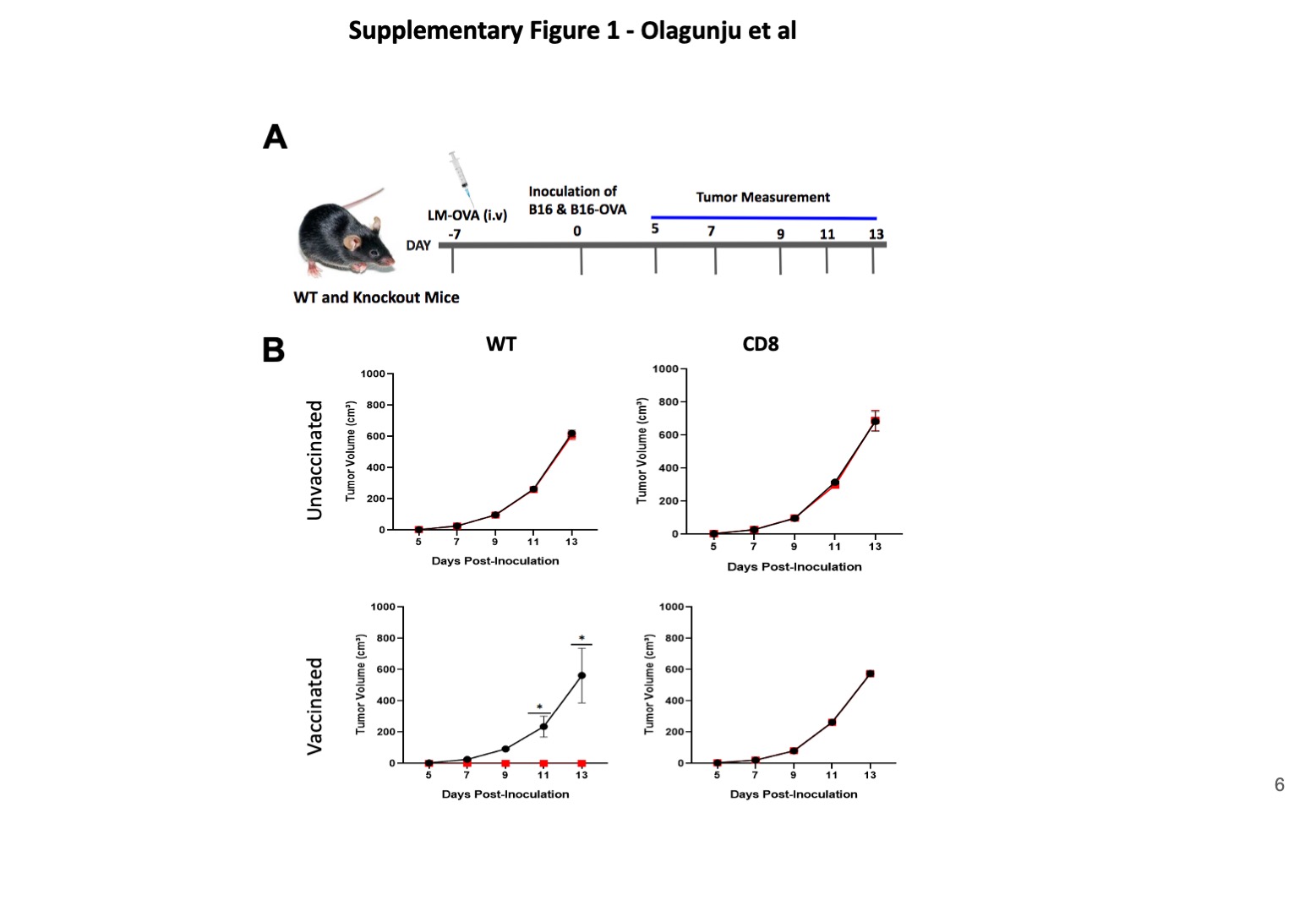

Supplement: Supplementary file 1 [file pathogens-13-00828-s001.zip › Olagunju et al Supplemental Figure 1.jpeg]

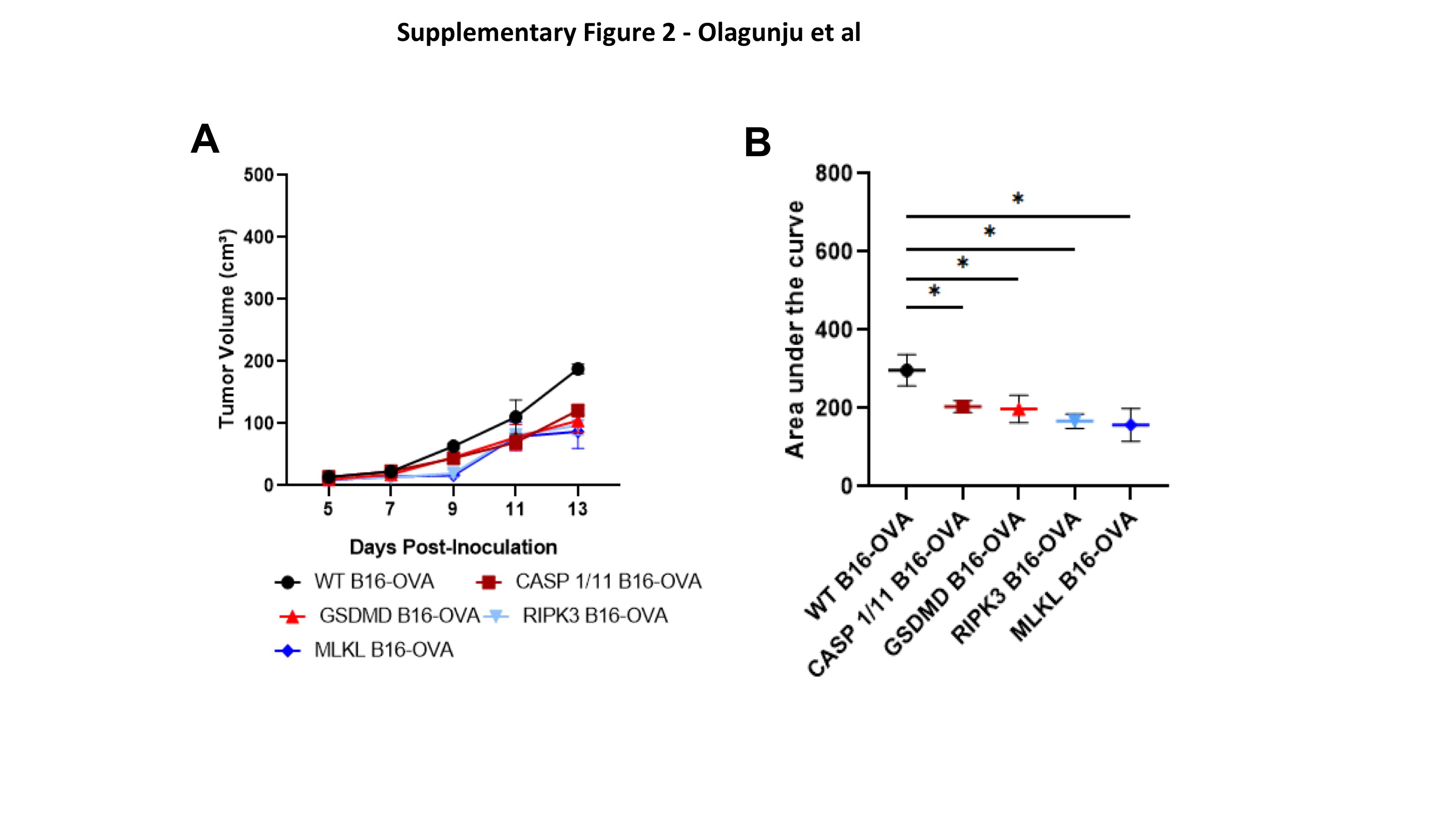

Supplement: Supplementary file 1 [file pathogens-13-00828-s001.zip › Olagunju et al Supplemental Figure 2.jpg]
